# Addendum: Kea show three signatures of domain-general statistical inference

**DOI:** 10.1038/s41467-020-16469-1

**Published:** 2020-06-04

**Authors:** Amalia P. M. Bastos, Alex H. Taylor

**Affiliations:** School of Psychology, The University of Auckland, Private Bag 92019, Auckland, 1142 New Zealand

**Keywords:** Evolution, Psychology, Animal behaviour

Addendum to: *Nature Communications* 10.1038/s41467-020-14695-1, published online 3 March 2020.

In the original version of the Article, our experimental and data processing methods did not account for the fact that tokens may have been visible between the experimenter’s fingers in some of the experimental trials. This could have provided kea with additional information when selecting between the jars in the experiment.

To rectify this issue, three independent coders first reviewed the video data files for all trials (*n* = 720) to identify the trials in which both experimenters’ hands were visible in the recording (*n* = 432). The video files for these trials were then reviewed using frame-by-frame analysis to identify trials in which token colours were visible to the kea. Coders identified 10 potentially problematic trials, out of the total 432 coded trials, where the subject may have had additional information prior to making their choice. These trials were then either substituted with the next available training trial (for kea that did not reach the 17/20 criteria in their first 20 trials), or removed entirely where no subsequent trials were available (for kea that did reach the 17/20 criteria in their first 20 trials). In the video data for the remaining 288 trials it was not possible to see both experimenters’ hands from an appropriate angle. Therefore, we randomly applied the observed error rate (10/432 trials, 2.31%) to these 288 trials, and so randomly allocated 7 trials out of these 288 to be either substituted by the next available trial within that condition, or removed entirely where subsequent trials were not available. The updated version of the original Table 1 is presented below as Table 1, which summarises our results following these changes.

**Table 1:** Individual performance in Experiments 1–3.

	Experiment 1 Condition 1	Experiment 1 Condition 2	Experiment 1 Condition 3	Experiment 2 Condition 1	Experiment 2 Condition 2	Experiment 3 Condition 1
Blofeld	10/20*** (BF = 0.27)	14/20** (BF = 1.29)	15/20** (BF = 3.22•)	18/20* (BF = 262.80•)	15/20** (BF = 3.22•)	12/20*** (BF = 0.40)
Bruce	12/20* (BF = 0.40)	15/20* (BF = 3.22•)	16/20* (BF = 10.31•)	17/20* (BF = 43.80•)	15/20* (BF = 3.22•)	17/20*** (BF = 43.80•)
Loki	15/20*** (BF = 3.22•)	16/20** (BF = 10.31•)	19/20*** (BF = 2496.61•)	18/20*** (BF = 262.80•)	18/19*** (BF = 1379.71•)	10/18*** (BF = 0.32)
Neo	14/20* (BF = 1.29)	14/20* (BF = 1.29)	15/20* (BF = 3.22•)	15/20* (BF = 3.22•)	14/20* (BF = 1.29)	15/20** (BF = 3.22•)
Plankton	15/20* (BF = 3.22•)	15/20** (BF = 3.22•)	16/20** (BF = 10.31•)	17/20* (BF = 43.80•)	17/20*** (BF = 43.80•)	9/19*** (BF = 0.28)
Taz	15/20*** (BF = 3.22•)	16/20* (BF = 10.31•)	17/20* (BF = 43.80•)	15/18* (BF = 16.91•)	16/20* (BF = 10.31•)	17/20*** (BF = 43.80•)

Number of correct trials performed by each subject (*n* = 6) within the first block of 20 trials for each condition of the three Experiments. In Experiment 3, correct trials constituted trials in which the subject chose the biased sampler, E1. Which of the two experimenters was the biased sampler (E1) was counterbalanced across subjects split into two groups: Neo, Bruce, Blofeld; and Loki, Plankton, Taz. Performance was tested using two-tailed Bayesian binomial tests (test value of 0.5, default Beta prior parameters at 1.0). Values with a Bayes Factor greater than 3 are followed by a dot. The seventeen blocks denoted by a single asterisk had all 20 trials manually verified by an independent coder. Seven further blocks, denoted by two asterisks, had at least 50% of all trials manually verified by an independent coder, and non-verified trials were included in the error simulation. Twelve further blocks, denoted by three asterisks, had all trials included in the error simulation.

To ensure that these changes did not affect the overall results of our study, we have re-run all statistical analyses affected by these changes. The new Bayesian binomial tests for individual performances are now provided (Table 1). The recoding and error simulation did not significantly affect the performance of any kea in any condition over the course of our three experiments. Our intercept-only model on first trial data reveals that the likelihood of a randomly selected kea succeeding in their first trial of any condition was 0.68 (pMCMC = 0.009), compared to a likelihood of 0.70 (pMCMC = 0.005) in the original analysis. In this updated dataset, subjects made the correct choice in 69.44% of all first trials. Considering only instances where the subject succeeded in that condition, performance was correct in 77.78% of first trials. As in the original results, re-analysis of the correlation between condition number and average performance within the first 20 trials of each condition did not reveal any evidence of learning effects (r = 0.151, BF = 0.509).

To check that our initial simulation was not an outlier, and so represents a likely set of substitutions or removals of seven randomly-assigned trials, we ran it an additional five times. In four of five of these repetitions, trial removals or substitutions did not affect subjects’ performance in any blocks: above chance performances remained above chance, and chance performances remained at chance. In one of the five repetitions, the simulation affected three blocks: one showed a performance change from chance levels to above chance, and two changed from above chance performance to chance. Following the changes, Blofeld showed above performance at 15/20 in Experiment 1’s Condition 2, and Plankton performed at 14/20 in the same condition, which, therefore, resulted in no difference to the total number of kea passing this test (4 of 6), and thus does not change our conclusions in any way. One of Neo’s correct trials in Experiment 3 was removed without substitution, reducing his score from 15/20 (BF = 3.22) to 14/19 (BF = 2.254) resulting in two, rather than three kea passing this condition. Thus only in one of the six simulations that we ran in total (one for our actual results, a further five to check the simulation robustness) did performance significantly change for any kea in any condition. Furthermore, even the one significant change we did observe across our six simulations does not affect our conclusions about kea intelligence, because a further two kea passed the condition in question.

In order to ensure that no other potential biases may have affected our data, we have run a new control experiment which addresses whether kea may have relied on any unintentional experimenter cues, such as body posture or breathing patterns, to select the hand holding the rewarding black token. We presented all six individuals included in the original study with twenty trials where both jars contained 55 rewarding tokens and 55 unrewarding tokens (50:50 ratio). In each set of trials, the experimenter consistently sampled a rewarding token from one jar, and an unrewarding token from the other. As in all test conditions, the experimenters wore mirrored sunglasses whilst sampling from the two jars. If subjects could use any unintentional experimenter cues or biases to choose the hand containing a rewarding black token, we would expect them to perform above chance across the twenty trials in this condition. On the other hand, if subjects were relying on the proportions of rewarding and unrewarding tokens to make their choices, they should perform at chance. None of the six kea performed above chance level at this control (Table 2).

**Table 2:** Individual performance in experiment controlling for unintentional experimenter cues or biases.

	Performance in new control Experiment
Blofeld	12/20 (BF = 0.396)
Bruce	13/20 (BF = 0.644)
Loki	9/20 (BF = 0.297)
Neo	8/20 (BF = 0.396)
Plankton	12/20 (BF = 0.396)
Taz	11/20 (BF = 0.297)

Number of correct trials performed by each subject (*n* = 6) within the first block of 20 trials for the experiment controlling for experimenter cues or biases. Performance was tested using two-tailed Bayesian binomial tests (test value of 0.5, default Beta prior parameters at 1.0). Values with a Bayes Factor lower than 0.3 provide strong support for the null hypothesis of no difference from chance.

Our updated results, alongside our new control condition, show the findings and conclusions of our original article are correct. Recoding and our initial error simulation did not significantly affect the performance of any kea for any condition of the original experiments, nor the results of our other analyses, while the simulation repetitions we ran show our initial simulation run was not an outlier. Finally, the additional control condition we report here finds no support for the hypothesis that the kea used any unintentional experimenter cues to guide their choices.

