# Supplementary material for: Kea show three signatures of domain-general statistical inference
Source: Nat Commun. 2020 Mar 3;11:828. doi: 10.1038/s41467-020-14695-1 (PMC7054307; doi:10.1038/s41467-020-14695-1)
Supplement: Supplementary file 1 — Supplementary Information [file 41467_2020_14695_MOESM1_ESM.pdf]

Supplementary Information

Subjects

Supplementary Table 1.

Subject	Age	Sex	Rearing History
Blofeld	5	Male	Captive bred
Bruce	6	Male	Wild
Loki	4	Male	Captive bred
Neo	6	Male	Captive bred
Plankton	4	Male	Captive bred
Taz	6	Male	Captive bred

Supplementary Table 1. All subjects were parent-reared. Ages (as of 2019) are estimated from hatch dates (captive bred subjects) or veterinarian evaluations (wild subjects).

Methods

Subjects and Apparatus

Our subjects were six male kea at Willowbank Wildlife Reserve (see Supplementary Table 1). Kea were housed in a large outdoor aviary, where food and water were available *ad libitum*. Participation in the study was voluntary and subjects were free to leave mid-session at any point. All subjects were trained to exchange rewarding (black) tokens and track human hands for previous experiments. Subjects were also previously trained to work on individual platforms.

Each subject was allocated an individual training platform (42cm x 42cm) within the aviary on which they were tested. Performance in trials was rewarded with soaked Science Hill Diet

pellets. A small wooden shelf (60cm x 20cm) with a plexiglass screen (43cm x 29cm) was used to separate subjects from the apparatus and the experimenter during testing. Transparent jars (⌀10.5cm, 16cm tall) were used during training and testing which contained populations of either rewarding (black) or unrewarding (orange) wooden tokens (7cm x 1cm x 1cm). Each jar held a maximum of 120 tokens. When the jars were too large for a population of tokens, tokens sat on a cardboard platform that was placed inside the jar, to ensure subjects could not see the experimenter's hands. Semi-circular cardboard lids (⌀11.5cm, 5.5cm tall) were attached to the top of each jar to ensure subjects could not see which tokens were being sampled. Where barriers were used, a blue foam disk (⌀10.5cm, 1cm thick) was added into the jar.

Initial Training

Throughout training and testing, subjects were required to select which of two closed hands contained an out-of-sight rewarding (black) token, while ignoring the hand containing the unrewarding (orange) token. Subjects indicated their preferred hand by touching it with their beak. The experimenter would then open the selected hand and hand over the rewarding token (if the subject selected the rewarding token) or wave and retreat the unrewarding token (if the subject made an incorrect choice). The rewarding token could then be exchanged with the experimenter for a food reward. Where subjects managed to grab the unrewarding token and attempted to exchange it, this was taken by the experimenter but not rewarded.

Subjects were trained to attend to and track hand trajectories for a previous study. Subjects were trained specifically for this study on a sampling motion, and making inferences about sampling from token populations in two jars, by selecting a hand that picked a rewarding token from a population of 100% rewarding tokens, over a hand that sampled from a population of 100% unrewarding tokens. In order to allow for a full counterbalancing of trial presentations

at test and minimise side biasing, subjects were also taught to simultaneously attend to the side on which jars were placed and whether hands were presented in parallel or crossed over. Trained was divided into four separate training steps, or phases, which are described below:

In Phase 1, subjects observed a sampling motion: the experimenter picked up a single token from a wooden shelf behind a plexiglass barrier and presented it to the kea within one of their fists. They then did the same motion with their other hand. One fist contained the rewarding (black) token, whilst the other contained the unrewarding (orange) token. This ensured the kea could track objects that were picked up and then held, out-of-sight in an experimenter's hand. Hands were presented either in parallel or crossed over each other. Subjects had to pass this stage of training at 17/20 to proceed to the next phase.

In Phase 2, subjects observed sampling from a population of tokens: the experimenter picked up a single token from either a transparent jar with 120 rewarding (black) tokens, or 120 unrewarding (orange) tokens. Kea could not see what the experimenter picked up, as the transparent jars had a cardboard lid which hid the experimenter's hands. Both jars were presented at the same time, though hands took turns sampling. The order of sampling was pseudorandomised. Jar positions remained the same for 10 consecutive trials at a time, and the experimenter's fists could be presented either in parallel or crossed over. This training step taught kea to attend to the contents of each jar and infer what could be sampled from them, i.e. only rewarding tokens from a 100% rewarding population, and only unrewarding tokens from a 100% unrewarding population. Subjects had to pass this stage of training at 17/20 to proceed to the next phase.

Phase 3 involved sampling from the same jars as before, one with 120 rewarding tokens and another with 120 unrewarding tokens, but now the experimenter's fists were only presented in parallel for every trial. The position of jars was pseudorandomised and counterbalanced within

blocks of 20 trials. This ensured that subjects attended to the fact that jar sides could be swapped regularly between trials. Only parallel hand motions were used in order to keep the new task demands to a bare minimum and facilitate learning. Subjects had to pass this stage of training at 17/20 to proceed to the next phase.

The final phase of training was identical to the previous stage, but now the experimenter's fists could be presented either in parallel or crossed over. Now, subjects were expected to synthesise all learning from previous steps together in a single demanding task. They had to attend to the contents of each jar, the position of each jar, and the trajectory followed by the experimenter's fists (parallel or crossed over) to succeed in any given trial. Experimenters wore mirrored sunglasses as a control for Clever Hans effects. To ensure that subjects were not attending to specific cues of a particular experimenter, subjects had to pass this stage of training at 17/20 with two different experimenters.

Once subjects passed all four phases of sampling training, they proceeded to Experiment 1.

Testing Procedures

Before each experimental session, subjects were given motivation trials, where they had to select and exchange a rewarding (black) token and ignore a nearby unrewarding (orange) token with the experimenter three times in a row, prior to the start of the session. This ensured subjects were eager to work and remembered which of the two tokens they should search for at test. Testing was carried out by three experimenters who were blind to experimental design and hypotheses, wearing mirrored sunglasses. Subjects only proceeded to the next testing condition or experiment upon reaching a criterion of 17/20 correct choices within the same block, or completing 240 trials (12 blocks) without reaching criterion. This ensured that subjects were confident in the current task before proceeding to a more demanding one.

Throughout testing, hand presentation (parallel or crossed), and location of the rewarding hand at time of choice were all pseudorandomised and counterbalanced within blocks of 20 trials.

Experiment 2 Training: Experiences with Physical Constraints to Sampling

Kea were given two training experiences before being tested for Experiment 2. The first one of these provided an egocentric learning opportunity, and the second provided an allocentric demonstration of the physical constraint imposed by a barrier.

In the first training experience, subjects were shown two small transparent jars (⌀6cm, 7.5cm tall), each containing 20 rewarding (black) tokens, where one had a blue foam barrier (1cm thick) at the top and the other did not. The jar with the barrier was therefore impossible to sample from, whereas the rewarding tokens in the open jar could be reached. Jars were presented by an experimenter for 5 seconds at a time, and sides were pseudorandomised. Subjects were allowed to touch either jar. If subjects touched the jar with the barrier, they were allowed to interact with the barrier for 3 seconds, then both jars were removed and the next trial was started immediately. Subjects learned to sample a black token from the accessible jar and were encouraged to exchange it for a food pellet with the experimenter. If subjects sampled more than one token, they were asked to exchange all their tokens before receiving a single food reward. This ensured that subjects were not motivated to take as many rewarding tokens from the accessible jar as possible within any given trial. Subjects had to touch the accessible jar first, and sample a rewarding token from it, on 17/20 trials in order to proceed to the next training experience.

In the second training experience, subjects watched as an experimenter sampled from two large jars (⌀10.5cm, 16cm tall) of 80 rewarding (black) tokens. One jar’s population was made inaccessible by a blue foam barrier (1cm thick) positioned above the tokens, whilst the other

did not contain a barrier. The experimenter attempted to sample from the inaccessible jar three times before closing their fist holding nothing, then with their other hand successfully sampled a rewarding token from the other jar. The order in which the two sampling demonstrations occurred was pseudorandomised and counterbalanced, as were the sides of the two jars. Both hands were then presented to the subject either in parallel or crossed over. As in previous training and testing, subjects indicated their chosen hand by touching the experimenter’s fist with their beak. If they found a rewarding (black) token, this was handed over and exchanged for a food reward. If they selected the closed fist, the experimenter opened their hands and showed them an empty palm, then proceeded to the next trial.

Raw Data

Supplementary Table 2. Individual Performance in Experiment 1

	Condition 1		Condition 2		Condition 3	
**Blofeld**	10/20<br>BF = 0.27	11	14/20<br>BF = 1.29	6	15/20<br>BF = 3.22	2
**Bruce**	12/20<br>BF = 0.40	6	16/20<br>BF = 10.31	6	16/20<br>BF = 10.31	4
**Loki**	15/20<br>BF = 3.22	5	16/20<br>BF = 10.31	2	19/20<br>BF = 2496.61	1
**Neo**	14/20<br>BF = 1.29	3	14/20<br>BF = 1.29	2	15/20<br>BF = 3.22	2
**Plankton**	15/20<br>BF = 3.22	8	15/20<br>BF = 3.22	2	16/20<br>BF = 10.31	4
**Taz**	15/20<br>BF = 3.22	3	16/20<br>BF = 10.31	2	17/20<br>BF = 43.80	1

Supplementary Table 2. Number of correct trials within the first block of each condition of Experiment 1. In grey, the number of blocks taken to reach 17/20 criterion, including the first block. Performance was tested using two-tailed Bayesian binomial tests (test value of 0.5, default Beta prior parameters at 1.0). Values with a Bayes Factor greater than 3 are highlighted in green.

Supplementary Table 3. Individual Performance in Experiment 2

	Condition 1		Condition 2	
**Blofeld**	18/20<br>BF = 262.80	1	15/20<br>BF = 3.22	4
**Bruce**	17/20<br>BF = 43.80	1	15/20<br>BF = 3.22	2
**Loki**	18/20<br>BF = 262.80	1	19/20<br>BF = 2496.61	1
**Neo**	15/20<br>BF = 3.22	3	14/20<br>BF = 1.29	4
**Plankton**	17/20<br>BF = 43.80	1	17/20<br>BF = 43.80	1
**Taz**	17/20<br>BF = 43.80	1	16/20<br>BF = 10.31	☒

Supplementary Table 3. Number of correct trials within the first block of each condition of Experiment 2. In grey, the number of blocks taken to reach 17/20 criterion, including first block. A crossed out grey block indicates 12 blocks without reaching criterion: Taz showed fluctuating performance and failed to pass criterion for Condition 2 in under 240 trials. Performance was tested using two-tailed Bayesian binomial tests (test value of 0.5, default Beta prior parameters at 1.0). Values with a Bayes Factor greater than 3 are highlighted in green.

Supplementary Table 4. Individual Performance in Experiment 3

	Distinguishing Experimenters	Preference Test	Demonstration	Probe Trials	Condition 1
**Blofeld**	20/20 (1)	10/20 (1)	9/20 (1)	18/20	12/20 BF = 0.40
**Bruce**	19/20 (1)	13/20 (2)	14/20 (1)	17/20	17/20 BF = 43.80
**Loki**	20/20 (1)	8/20 (3)	15/20 (1)	18/20	11/20 BF = 0.30
**Neo**	19/20 (1)	11/20 (1)	7/20 (2)	19/20	15/20 BF = 3.22
**Plankton**	20/20 (1)	12/20 (2)	14/20 (1)	18/20	10/20 BF = 0.27
**Taz**	19/20 (1)	10/20 (1)	12/20 (1)	18/20	17/20 BF = 43.80

Supplementary Table 4. Number of trials in which the subject made the correct choice (in the first and fourth columns) or chose the biased sampler (all other columns) in each block. In grey, number of blocks taken to reach criterion, including the first block. Experimenter identification (column 1) and probe trials (column 4) both had the same training criterion of 17/20. For the preference test (column 2), criterion was between 9/20 and 11/20. For the demonstration phase (column 3), criterion was the choice of the biased sampler (E1) at 9/20 or above (i.e. not showing a preference for E2). Which of the two experimenters was the biased sampler (E1) was counterbalanced across subjects split into two groups: Neo, Bruce, Blofeld; and Loki, Plankton, Taz. Loki did not experience three trials of pre-test demonstration before being tested, due to experimenter error. Performance was tested using two-tailed Bayesian binomial tests (test value of 0.5, default Beta prior parameters at 1.0). Values with a Bayes Factor greater than 3 are highlighted in green. All Bayes Factor values are provided, rounded to two decimal places.

Supplementary Table 5. Learning Effects Analyses within Conditions

		r	Bayes Factor
**Experiment 1**	**Condition 1**	0.019	0.278
	**Condition 2**	-0.275	0.527
	**Condition 3**	-0.157	0.339
**Experiment 2**	**Condition 1**	0.335	0.733
	**Condition 2**	-0.371	0.928
**Experiment 3**	**Condition 1**	0.174	0.357

Supplementary Table 5. Results of correlation Bayesian correlation tests (non-directional correlation, prior width = 1) of trial number and average performance across all subjects, for each of the conditions tested across the three experiments. A Bayes factor (BF) < 0.33 shows substantial support for the null hypothesis, whilst a BF > 3 shows substantial support for the competing hypothesis.

Annotated R Code

R code used to analyse learning effects across conditions and first trial performance data.

Learning Effects Analysis

```
d <- read.csv("~Data_KeaIS_FirstTrialPerformance_1-0.csv")
d$condition <- as.factor(d$condition) ## save condition as a factor
```

```
d
##          ID experiment condition successes
## 1   Blofeld          1         1         0
## 2   Blofeld          1         2         0
## 3   Blofeld          1         3         1
## 4   Blofeld          2         4         1
## 5   Blofeld          2         5         1
## 6   Blofeld          3         6         0
## 7     Bruce          1         1         0
## 8     Bruce          1         2         1
## 9     Bruce          1         3         1
## 10    Bruce          2         4         1
## 11    Bruce          2         5         0
## 12    Bruce          3         6         1
## 13     Loki          1         1         1
## 14     Loki          1         2         1
## 15     Loki          1         3         1
## 16     Loki          2         4         0
## 17     Loki          2         5         1
## 18     Loki          3         6         1
## 19      Neo          1         1         0
## 20      Neo          1         2         1
## 21      Neo          1         3         1
## 22      Neo          2         4         1
## 23      Neo          2         5         1
## 24      Neo          3         6         0
## 25 Plankton          1         1         0
## 26 Plankton          1         2         1
## 27 Plankton          1         3         1
## 28 Plankton          2         4         1
## 29 Plankton          2         5         1
## 30 Plankton          3         6         1
## 31      Taz          1         1         1
## 32      Taz          1         2         1
## 33      Taz          1         3         0
## 34      Taz          2         4         1
## 35      Taz          2         5         1
## 36      Taz          3         6         1
```

First Trial Performance Model

```
## intercept-only null Bayesian model using Monte-Carlo estimates

m1.1 <- brm(data = d, family = bernoulli,
            successes ~ 0 + intercept,
            prior = prior(normal(0, 1), class = b))


## Compiling the C++ model
## Start sampling
##
## SAMPLING FOR MODEL 'a3e4e4799f2e82fe85c4ad29b33001c6' NOW (CHAIN 1).
## Chain 1:
## Chain 1: Gradient evaluation took 0 seconds
## Chain 1: 1000 transitions using 10 leapfrog steps per transition would
take 0 seconds.
## Chain 1: Adjust your expectations accordingly!
## Chain 1:
## Chain 1:
## Chain 1: Iteration:    1 / 2000 [  0%]  (Warmup)
## Chain 1: Iteration:  200 / 2000 [ 10%]  (Warmup)
## Chain 1: Iteration:  400 / 2000 [ 20%]  (Warmup)
## Chain 1: Iteration:  600 / 2000 [ 30%]  (Warmup)
## Chain 1: Iteration:  800 / 2000 [ 40%]  (Warmup)
## Chain 1: Iteration: 1000 / 2000 [ 50%]  (Warmup)
## Chain 1: Iteration: 1001 / 2000 [ 50%]  (Sampling)
## Chain 1: Iteration: 1200 / 2000 [ 60%]  (Sampling)
## Chain 1: Iteration: 1400 / 2000 [ 70%]  (Sampling)
## Chain 1: Iteration: 1600 / 2000 [ 80%]  (Sampling)
## Chain 1: Iteration: 1800 / 2000 [ 90%]  (Sampling)
## Chain 1: Iteration: 2000 / 2000 [100%]  (Sampling)
## Chain 1:
## Chain 1:  Elapsed Time: 0.043 seconds (Warm-up)
## Chain 1:                0.05 seconds (Sampling)
## Chain 1:                0.093 seconds (Total)
## Chain 1:
##
## SAMPLING FOR MODEL 'a3e4e4799f2e82fe85c4ad29b33001c6' NOW (CHAIN 2).
## Chain 2:
## Chain 2: Gradient evaluation took 0 seconds
## Chain 2: 1000 transitions using 10 leapfrog steps per transition would
take 0 seconds.
## Chain 2: Adjust your expectations accordingly!
## Chain 2:
## Chain 2:
## Chain 2: Iteration:    1 / 2000 [  0%]  (Warmup)
## Chain 2: Iteration:  200 / 2000 [ 10%]  (Warmup)
## Chain 2: Iteration:  400 / 2000 [ 20%]  (Warmup)
## Chain 2: Iteration:  600 / 2000 [ 30%]  (Warmup)
## Chain 2: Iteration:  800 / 2000 [ 40%]  (Warmup)
## Chain 2: Iteration: 1000 / 2000 [ 50%]  (Warmup)
## Chain 2: Iteration: 1001 / 2000 [ 50%]  (Sampling)
## Chain 2: Iteration: 1200 / 2000 [ 60%]  (Sampling)
## Chain 2: Iteration: 1400 / 2000 [ 70%]  (Sampling)
## Chain 2: Iteration: 1600 / 2000 [ 80%]  (Sampling)
## Chain 2: Iteration: 1800 / 2000 [ 90%]  (Sampling)
## Chain 2: Iteration: 2000 / 2000 [100%]  (Sampling)
## Chain 2:
## Chain 2:  Elapsed Time: 0.034 seconds (Warm-up)
```

```
## Chain 2:                0.051 seconds (Sampling)
## Chain 2:                0.085 seconds (Total)
## Chain 2:
##
## SAMPLING FOR MODEL 'a3e4e4799f2e82fe85c4ad29b33001c6' NOW (CHAIN 3).
## Chain 3:
## Chain 3: Gradient evaluation took 0 seconds
## Chain 3: 1000 transitions using 10 leapfrog steps per transition would
take 0 seconds.
## Chain 3: Adjust your expectations accordingly!
## Chain 3:
## Chain 3:
## Chain 3: Iteration:    1 / 2000 [  0%]  (Warmup)
## Chain 3: Iteration:  200 / 2000 [ 10%]  (Warmup)
## Chain 3: Iteration:  400 / 2000 [ 20%]  (Warmup)
## Chain 3: Iteration:  600 / 2000 [ 30%]  (Warmup)
## Chain 3: Iteration:  800 / 2000 [ 40%]  (Warmup)
## Chain 3: Iteration: 1000 / 2000 [ 50%]  (Warmup)
## Chain 3: Iteration: 1001 / 2000 [ 50%]  (Sampling)
## Chain 3: Iteration: 1200 / 2000 [ 60%]  (Sampling)
## Chain 3: Iteration: 1400 / 2000 [ 70%]  (Sampling)
## Chain 3: Iteration: 1600 / 2000 [ 80%]  (Sampling)
## Chain 3: Iteration: 1800 / 2000 [ 90%]  (Sampling)
## Chain 3: Iteration: 2000 / 2000 [100%]  (Sampling)
## Chain 3:
## Chain 3:  Elapsed Time: 0.04 seconds (Warm-up)
## Chain 3:                0.046 seconds (Sampling)
## Chain 3:                0.086 seconds (Total)
## Chain 3:
##
## SAMPLING FOR MODEL 'a3e4e4799f2e82fe85c4ad29b33001c6' NOW (CHAIN 4).
## Chain 4:
## Chain 4: Gradient evaluation took 0 seconds
## Chain 4: 1000 transitions using 10 leapfrog steps per transition would
take 0 seconds.
## Chain 4: Adjust your expectations accordingly!
## Chain 4:
## Chain 4:
## Chain 4: Iteration:    1 / 2000 [  0%]  (Warmup)
## Chain 4: Iteration:  200 / 2000 [ 10%]  (Warmup)
## Chain 4: Iteration:  400 / 2000 [ 20%]  (Warmup)
## Chain 4: Iteration:  600 / 2000 [ 30%]  (Warmup)
## Chain 4: Iteration:  800 / 2000 [ 40%]  (Warmup)
## Chain 4: Iteration: 1000 / 2000 [ 50%]  (Warmup)
## Chain 4: Iteration: 1001 / 2000 [ 50%]  (Sampling)
## Chain 4: Iteration: 1200 / 2000 [ 60%]  (Sampling)
## Chain 4: Iteration: 1400 / 2000 [ 70%]  (Sampling)
## Chain 4: Iteration: 1600 / 2000 [ 80%]  (Sampling)
## Chain 4: Iteration: 1800 / 2000 [ 90%]  (Sampling)
## Chain 4: Iteration: 2000 / 2000 [100%]  (Sampling)
## Chain 4:
## Chain 4:  Elapsed Time: 0.045 seconds (Warm-up)
## Chain 4:                0.04 seconds (Sampling)
## Chain 4:                0.085 seconds (Total)
## Chain 4:
```

Note that the `echo = FALSE` parameter was added to the code chunk to prevent printing of the R code that generated the plot.

```
save(m1.1, file = 'm1.1.rda')
```

```
print(m1.1)
##  Family: bernoulli
##   Links: mu = logit
## Formula: successes ~ 0 + intercept
##    Data: d (Number of observations: 36)
## Samples: 4 chains, each with iter = 2000; warmup = 1000; thin = 1;
##          total post-warmup samples = 4000
##
## Population-Level Effects:
##           Estimate Est.Error l-95% CI u-95% CI Eff.Sample Rhat
## intercept     0.85      0.34     0.20     1.55       1316 1.00
##
## Samples were drawn using sampling(NUTS). For each parameter, Eff.Sample
## is a crude measure of effective sample size, and Rhat is the potential
## scale reduction factor on split chains (at convergence, Rhat = 1).

plot(m1.1)
```

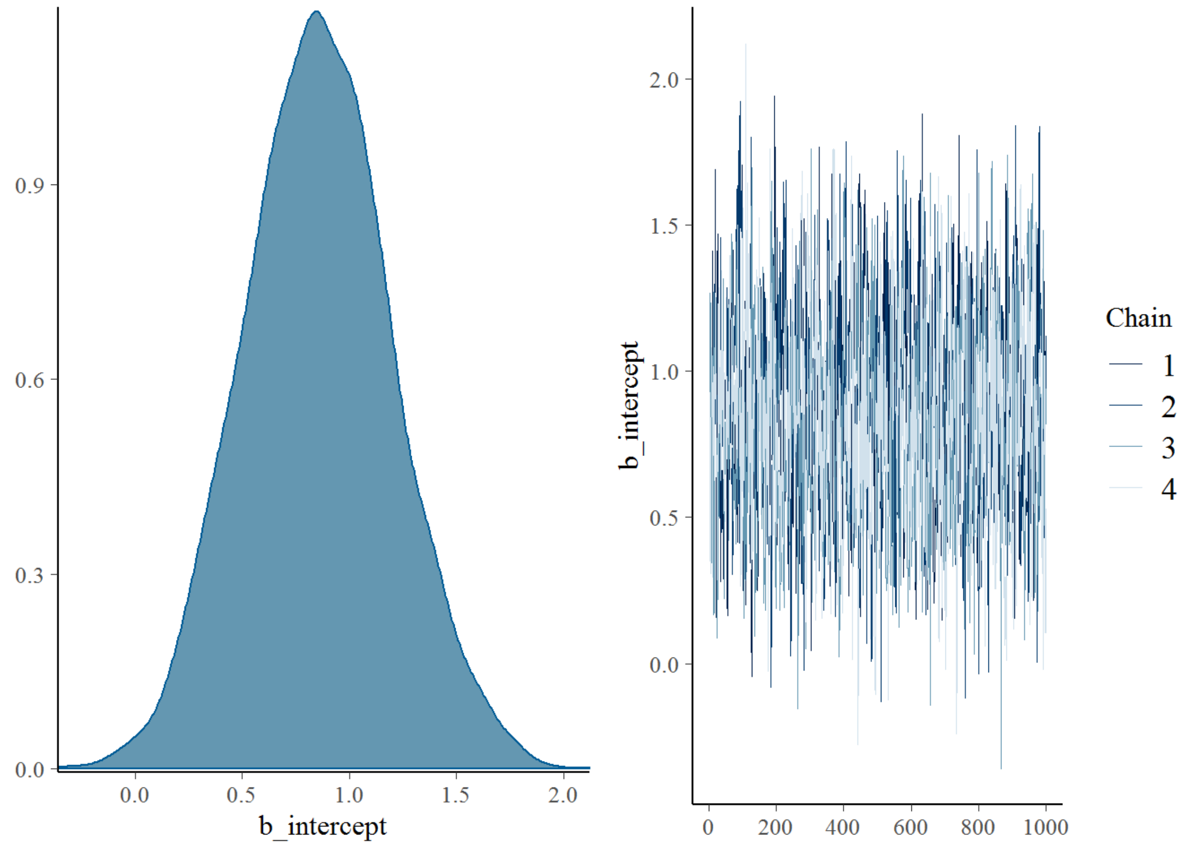


```
prior_summary(m1.1)
##           prior class      coef group resp dpar nlpar bound
## 1 normal(0, 1)     b
## 2                  b intercept

post1 <- posterior_samples(m1.1)

## likelihood of success by the average kea, across all trials and
experiments, in their first performance

p.1 <- inv_logit_scaled(post1$b_intercept)

hist(p.1)
```

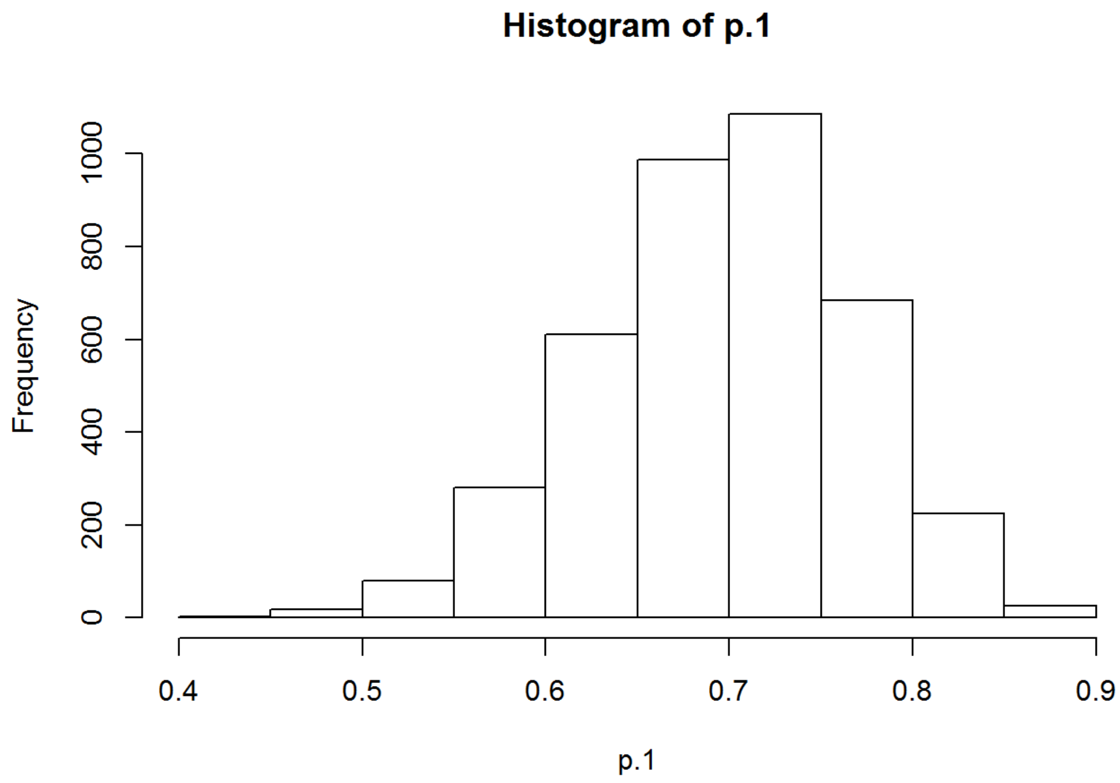


```
median(p.1)
## [1] 0.7005451
sum(p.1 < 1/2) / 4000
## [1] 0.00525
```
