# Supplementary material for: Kea show three signatures of domain-general statistical inference
Source: Nat Commun. 2020 Mar 3;11:828. doi: 10.1038/s41467-020-14695-1 (PMC7054307; doi:10.1038/s41467-020-14695-1)
Supplement: Supplementary file 3 — Reporting Summary [file 41467_2020_14695_MOESM3_ESM.pdf]

nature research

Corresponding author(s): Amalia P M Bastos

Last updated by author(s): 22-01-2020

Reporting Summary

Nature Research wishes to improve the reproducibility of the work that we publish. This form provides structure for consistency and transparency in reporting. For further information on Nature Research policies, see Authors & Referees and the Editorial Policy Checklist .

Statistics

For all statistical analyses, confirm that the following items are present in the figure legend, table legend, main text, or Methods section.

n/a	Confirmed	
☐	☒	The exact sample size (*n*) for each experimental group/condition, given as a discrete number and unit of measurement
☐	☒	A statement on whether measurements were taken from distinct samples or whether the same sample was measured repeatedly
☐	☒	The statistical test(s) used AND whether they are one- or two-sided<br>*Only common tests should be described solely by name; describe more complex techniques in the Methods section.*
☒	☐	A description of all covariates tested
☒	☐	A description of any assumptions or corrections, such as tests of normality and adjustment for multiple comparisons
☐	☒	A full description of the statistical parameters including central tendency (e.g. means) or other basic estimates (e.g. regression coefficient) AND variation (e.g. standard deviation) or associated estimates of uncertainty (e.g. confidence intervals)
☒	☐	For null hypothesis testing, the test statistic (e.g. *F*, *t*, *r*) with confidence intervals, effect sizes, degrees of freedom and *P* value noted<br>*Give P values as exact values whenever suitable.*
☐	☒	For Bayesian analysis, information on the choice of priors and Markov chain Monte Carlo settings
☒	☐	For hierarchical and complex designs, identification of the appropriate level for tests and full reporting of outcomes
☒	☐	Estimates of effect sizes (e.g. Cohen's *d*, Pearson's *r*), indicating how they were calculated

Our web collection on statistics for biologists contains articles on many of the points above.

Software and code

Policy information about availability of computer code

Data collection	N/A
Data analysis	JASP 0.9.2, R 3.4.1, Stan

For manuscripts utilizing custom algorithms or software that are central to the research but not yet described in published literature, software must be made available to editors/reviewers. We strongly encourage code deposition in a community repository (e.g. GitHub). See the Nature Research guidelines for submitting code & software for further information.

Data

Policy information about availability of data

All manuscripts must include a data availability statement. This statement should provide the following information, where applicable:

- Accession codes, unique identifiers, or web links for publicly available datasets
- A list of figures that have associated raw data
- A description of any restrictions on data availability

Our full dataset is available in Supplementary Data 1.

Field-specific reporting

Please select the one below that is the best fit for your research. If you are not sure, read the appropriate sections before making your selection.

☐ Life sciences ☒ Behavioural & social sciences ☐ Ecological, evolutionary & environmental sciences

For a reference copy of the document with all sections, see nature.com/documents/nr-reporting-summary-flat.pdf

Behavioural & social sciences study design

All studies must disclose on these points even when the disclosure is negative.

Study description	The study involved quantitative data in a within-subjects design. Responses were coded as either correct or incorrect.
Research sample	Six male captive kea (Nestor notabilis) aged between 4 and 6 years. The sample is representative of the cognitive capacity of an adult male kea. Subjects were self-selected, as participation was voluntary: we tested only the subjects that wished to participate in the study.
Sampling strategy	All subjects (n=6) performed all experimental tasks. Trial types were pseudorandomised (no more than 2 of the same type in a row) and counterbalanced within blocks of 20 trials. No sample size calculation was performed, as participation was fully voluntary.
Data collection	Data collection was performed in situ (written down with pen and paper by experimenters blind to conditions and hypothesis, and video-recorded for blind coding). Two or more experimenters were present within the enclosure at all times. Three experimenters blind to experimental conditions and study hypotheses collected all data for Experiments 1 and 2. One non-blind experimenter assisted with data collection for Experiment 3, but their role was randomised and counterbalanced between individuals. One further experimenter blind to experimental conditions and study hypotheses coded 10% of video data.
Timing	Training and data collection took place between 21 January 2019 and 12 April 2019.
Data exclusions	No data was excluded from any of our analyses.
Non-participation	All 13 kea present in the aviary were given the opportunity to take part in the training required for participation in the study. Only seven participants were sufficiently motivated to complete all the training necessary to participate in the experiment. One of the seven kea lost motivation to participate in the experiment halfway through Condition 2 of Experiment 1, leaving us with a final sample size of six kea, that completed all the experiments.
Randomization	All aspects of testing conditions (hand trajectory, order of sampling) were randomized and counterbalanced within blocks of 20 trials for all experiments. Between-subject randomization was only required for Experiment 3. During this experiment, which of two experimenters was biased (E1) or unbiased (E2) was randomized between subjects.

Reporting for specific materials, systems and methods

We require information from authors about some types of materials, experimental systems and methods used in many studies. Here, indicate whether each material, system or method listed is relevant to your study. If you are not sure if a list item applies to your research, read the appropriate section before selecting a response.

Materials & experimental systems

n/a	Involved in the study	
☒	☐	Antibodies
☒	☐	Eukaryotic cell lines
☒	☐	Palaeontology
☐	☒	Animals and other organisms
☒	☐	Human research participants
☒	☐	Clinical data

Methods

n/a	Involved in the study	
☒	☐	ChIP-seq
☒	☐	Flow cytometry
☒	☐	MRI-based neuroimaging

Animals and other organisms

Policy information about studies involving animals; ARRIVE guidelines recommended for reporting animal research

Laboratory animals	Six captive kea (Nestor notabilis) housed at Willowbank Wildlife Reserve were tested for this study. All subjects were male and aged between 4 and 6 years.
Wild animals	*Provide details on animals observed in or captured in the field; report species, sex and age where possible. Describe how animals were caught and transported and what happened to captive animals after the study (if killed, explain why and describe method; if released, say where and when) OR state that the study did not involve wild animals.*
Field-collected samples	*For laboratory work with field-collected samples, describe all relevant parameters such as housing, maintenance, temperature, photoperiod and end-of-experiment protocol OR state that the study did not involve samples collected from the field.*
Ethics oversight	Research was conducted under ethics approval from The University of Auckland Ethics Committee (reference number 001816).

Note that full information on the approval of the study protocol must also be provided in the manuscript.
