# Supplementary material for: Kea show three signatures of domain-general statistical inference
Source: Nat Commun. 2020 Mar 3;11:828. doi: 10.1038/s41467-020-14695-1 (PMC7054307; doi:10.1038/s41467-020-14695-1)
Supplement: Supplementary file 4 — Description of Additional Supplementary Files [file 41467_2020_14695_MOESM4_ESM.pdf]

Description of Additional Supplementary Files

File Name: Supplementary Data 1
Description: Individual data for all subjects is provided for both test trials and between-test training trials.
